# Seasonal variation of lupus nephritis in a cohort of Egyptian patients

**DOI:** 10.1007/s10067-022-06442-2

**Published:** 2022-11-16

**Authors:** Tarek Samy Abdelaziz, Nehal K. Rakha, Tarek Fayad, Geilan A. Mahmoud, Ahmed Fayed, Hany Hammad

**Affiliations:** 1 Department of Renal Medicine, KasrAlainy University Hospitals-Cairo University, AlSaraystreet, El Manial, Cairo, Egypt; 2 Department of Rheumatology, Cairo University Hospitals, Cairo, Egypt

**Keywords:** Lupus erythematosus, Lupus nephritides, Lupus nephritis, Seasonal variation

## Abstract

**Introduction:**

Systemic lupus erythematosus is an autoimmune multisystem disease; renal affection is one of its most common manifestations. The effect of environmental factors on lupus nephritis flares is not fully understood.

**Methods:**

This is a retrospective study that included 200 patients with lupus nephritis flares. All patients had confirmed diagnosis of lupus nephritis on histopathological examination. Lupus nephritis flares were defined by either (1) nephritic flare: defined as increased proteinuria or serum creatinine concentration; abnormal urinary sediment or a reduction in creatinine clearance, or (2) proteinuria flare defined as persistent increase in proteinuria > 0.5–1.0 g/day after achieving complete remission; doubling to > 1 g/day after achieving partial remission. The time of renal flare (month of the year) was recorded to determine the effect of seasonal variation on lupus nephritis flares.

**Results:**

The median age for the patients was 33 years (IQR = 13); 92% of patients were females. The median duration of lupus was 7 years (IQR = 6). The median serum creatinine was 1.4 mg/dl, median serum urea level was 32, and median UPCR was 2.4 gm/dl. The highest incidence of flares occurred in June (14%) and July (12.5%) (*p* = 0.003).

**Conclusion:**

Seasonal pattern of LN flare was observed in our study in Egyptian cohort of patients, with most flares observed during meteorological summertime. Larger studies are needed to confirm this seasonal pattern.

**Table:** 

**Key Points** *• Flares of lupus nephritis are common in patients with systemic lupus erythromatosus.* *• A seasonal pattern of flares of lupus nephritis was observed in our study. This seasonal pattern has been observed by previous studies in variable ethnicities and variable climatic circumstances.*

## Introduction

Systemic lupus erythematosus (SLE) is an autoimmune disease characterized by multi-organ affection [1]. The exact risk factors, etiology, pathogenesis, and optimum treatment modalities are not fully understood as knowledge about SLE is evolving. The disease is characterized by remitting and exacerbating flares that affect multiple organs of the body, either simultaneously or sequentially. Studies observing the incidence and prevalence of SLE revealed variations regarding age, sex, ethnicity, and time among different areas worldwide [2–8]. The disease can affect many organs by autoantibodies against nuclear and cytoplasmic antigens. It is postulated that autoantibodies start to accumulate years before the diagnosis. Other contributing factors for tissue damage in SLE are defective cell death and defective elimination of dying cells, leading to the generation of autoantibodies that stimulate inflammatory and immune responses. SLE is described as multi-factorial in nature; many factors are associated with the disease development as genetic factors, ethnic, immunologic, hormonal, and environmental factors [9–14]. Environmental factors like ultraviolet rays “exposure, smoking, drugs, and viruses” were hypothesized to be involved in triggering SLE disease activity, in genetically predisposed people. The recent climate changes in the form of greenhouse gas emission and global warming stand significant health hazards. Important international reports project that, by 2050, the climate changes will have health impacts; patients with climate sensitive diseases are most vulnerable [15]. Renal system is one of the most commonly affected systems throughout the course of lupus activity [16]. Renal involvement may be early or late presentation during the disease course [17]. Renal involvement may be minimal with no evident clinical manifestations or may only be detected by investigations. Clinical presentations of LN vary widely from asymptomatic disease only detectable by laboratory investigations to proteinuria with its clinical manifestations ranging from mild degree to nephrotic range protenuria, or may even manifest by nephritic syndrome to a more severe form of renal failure [18].

**Fig. 1:**
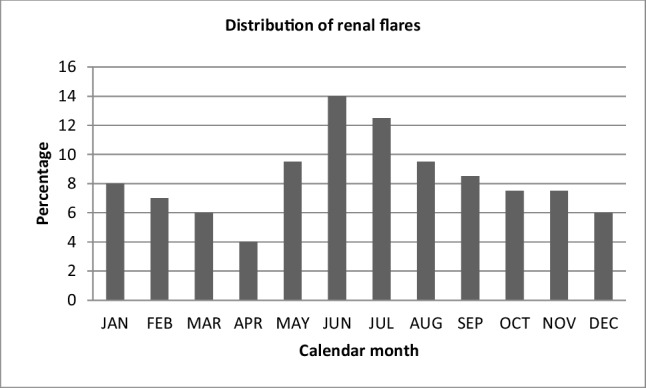
Distribution of renal flares per calendar month

Diagnosis and identification of renal flares are essential to improve outcome (Fig. 1). Environmental factors have recently been investigated as possible triggers for LN flares. If causal association is established, this would help decrease LN flares and hence improve patients’ outcome.

## Methodology and patients’ selection

### Study design

This study was a retrospective study. Records of 753 patients were reviewed between 2005 and 2015. Two hundred patients with lupus nephritis flare were included in this study. Patients were being followed up in the rheumatology and nephrology outpatient department. For the simplicity of analysis, each patient had only one entry which is the first flare of lupus nephritis.

### Ethical approval and study oversight

The study protocol was approved by the KasrAlainy Research Ethics Committee approval number KA20-117 M. The study adhered to the principles of Declaration of Helsinki.

### Data collection

Electronic and paper records of patients were reviewed, and data were extracted.

### Eligibility criteria

Entry criteria for this study were the pathological and serological diagnosis of lupus nephritis (LN). Other organ involvement of SLE was recorded. LN duration and treatment regimen were obtained.

### Establishing the diagnosis of systemic lupus erythromatosus

We revised the data of patients diagnosed with SLE according to 1997 ACR Revised Criteria for Classification of SLE [19]. Each patient had at least 4 confirmed criteria out of 11 defined by ACR for classification of SLE.

### Establishing the diagnosis of lupus nephritis

All patients had confirmed diagnosis of lupus nephritis on histopathological examination or percutaneous kidney biopsy specimens. Renal biopsies were performed by percutaneous ultrasound guided technique as it is the standard of care [20]; all were examined under light microscopy using immune staining techniques. The biopsies were done by a well-trained nephrologists or interventional radiologists.

Histopathological classification of renal biopsies was performed according to ISN/RP which classified LN histopathology into 6 classes depending on findings by LM.

Class I (minimal mesangial LN)

Class II (mesangial proliferative LN)

Class III (focal LN) which could be active (A), active and chronic lesions (A/C), or chronic inactive lesions (C)

Class IV (diffuse LN) which is further classified into IV-S(A) active lesions; diffuse segmental proliferative LN, IV-G(A): active lesions; diffuse global proliferative LN, IV-S(A/C): active and chronic lesions; diffuse segmental proliferative and sclerosing LN or IV-G(A/C): active and chronic lesions; diffuse global proliferative

Class V (membranous LN)

Class VI (advanced sclerotic LN) [16]

### Definition of renal flares

A renal flare was defined as either (1) nephritic flare: defined as increased proteinuria or serum creatinine concentration; abnormal urinary sediment or a reduction in creatinine clearance due to the presence of active disease; increase or recurrence of active urinary sediment, with or without proteinuria; usually associated with decrease in renal function, or severe increase or recurrence of active urinary sediment with or without increase in proteinuria; ≥ 25% increase serum creatinine concentration or (2) proteinuric flare: defined as persistent increase in proteinuria > 0.5–1.0 g/day after achieving complete remission; doubling to > 1 g/day after achieving partial remission.

Patients’ laboratory profile including erythrocyte sedimentation rate (ESR), anti-dsDNA antibodies, serum urea and creatinine, urine analysis, and 24-h urinary proteins at the time of lupus nephritis flare were obtained.

### Urine sample analysis

Urine samples were examined for physical and chemical properties. Microscopic examination for pus cells, red blood cells, epithelial cells, casts, bacteria, and others like uric acid and calcium oxalates.

### Quantification of proteinuria

We collected data of microscopic examination coupled with 24-h urinary proteins for assessment for LN flare. 24-h urinary protein collection was done for each patient by collecting urine in one or more containers over a period of 24 h. The patients were instructed to collect urine starting from second void in the morning and for 24 h till next day morning. The containers were kept in a cool environment and then sent to lab for analysis.

### Determination of seasonal variations of lupus nephritis

A season is defined as a division of the year based on changes in weather, ecology, and the number of daylight hours in a given region. In Egypt, winter generally begins on December 21 or 22. This is the winter solstice, the day of the year with the shortest period of daylight. Summer begins on June 20 or 21, the summer solstice, which has the most daylight of any day in the year. Spring and fall, or autumn, begin on equinoxes, days that have equal amounts.

### Systemic Lupus Erythematosus Disease Activity Index 2000

This is a well-validated score to measure lupus activity. We have calculated this score retrospectively using data extracted from patients’ records [21].

### Cutaneous Lupus Disease Area and Severity Index

We have applied this score to measure the skin disease activity. This score was developed by American dermato-rheumatologists and has been validated in many studies. The score has a domain for measuring the active skin disease [22].

### Seasons dates in Egypt

Hours of sunshine and median temperature are shown in Table 1 [23]. Astronomical seasons are demarcated in Egypt as follows [24]. Spring starts with spring equinox which is 20th of March. Summer starts with summer solstice which is the 21st of June. Autumn starts with autumn equinox which is 23rd of September. Winter starts with winter solstice which is 21st of December.

**Table 1:** Median temperature and hours of sunshine in Egypt by month

Month	Median (range)	Hours of sunshine
January	13.71 (6.82–20.66)	10.28
February	15.21 (7.85–22.61)	11.11
March	18.73 (11.21–26.31)	12.01
April	23.23 (15.26–31.25)	12.56
May	27.1 (19.32–34.93)	13.42
June	29.63 (21.98–73.32)	14.07
July	30.53 (23.39–73.72)	13.57
August	30.58 (23.43–37.77_	13.17
September	28.67 (21.67–35.71)	12.24
October	25.12 (18.1–32.2)	11.3
November	19.52 (12.64–26.45)	10.41
December	15.13 (8.26–22.04)	10.16

According to meteorological definition, the season starts with the first day of the month that has the equinox or solstice which means that summer starts from 1st of June till 31st of August.

## Results

The median age for the patients was 33 years (IQR = 13); 92% of patients were females. The median duration of lupus was 7 years (IQR = 6). The median serum creatinine was 1.4 mg/dl, median serum urea level was 32, and median UPCR was 2.4 gm/dl as shown in Table 2. Frequencies of systemic manifestations of SLE are shown in Table 3. Arthritis is the most common systemic manifestation, followed by cutaneous manifestations. The type of renal flares (nephritic or proteinuria) is shown in Table 4. Classes of lupus nephritis are shown in Table 5.

**Table 2:** Baseline patients characteristics

Age years, median (IQR)	33 (13)
Duration of lupus years median (IQR)	7 (6)
Sex, female number (%)	184 (92%)
Serumurea	32 (27)
Serum creatinine	1.4 (0.9)
UPCR	2.4 (2.38)

**Table 3:** Frequencies of systemic manifestations

	Frequency	Percentage
Cutaneous	133	66.5%
Arthritic	154	77%
Serositis	45	22.5%
Neurological	4	2%
Hematological	64	32%
Constitutional	47	23.5%

**Table 4:** Breakdown of the type of renal flare in our cohort

Type of flare	Percent
Nephritic flares	74%
Proteinuria flare	72%

**Table 5:** Frequencies of lupus nephritis classes

Lupus nephritis class	Frequency	Percent
II	9	4.5%
III	84	42%
IV	88	44%
V	19	9.5%
Total	200	100

Maintenance regimens for lupus nephritis are shown in Table 6. Distribution of flares by month is shown in Table 7. The highest frequency of flares was in June followed by July.

**Table 6:** Maintenance treatment regimen for lupus nephritis

Regimen	Frequency	Percentage
Prednisone-AZA-HCQ	179	90%
Prednisone-MMF-HCQ	21	10%

**Table 7:** Distribution of flares by month

Month	Frequency	Percent (%)
January	16	8.0
February	14	7.0
March	12	6.0
April	8	4.0
May	**19**	9.5
June	28	14
July	25	12.5
August	19	9.5
September	17	8.5
October	15	7.5
November	15	7.5
December	12	6.0
Total	200	100.0

### SLEDAI-2 K score and activity score skin activity score

The median and range of both scores is shown in Table 8. There was statistically significant differences between calendar months. Higher scores were observed during the summertime.

**Table 8:** SLEDAI-2 K SCORE and skin activity score by calendar month

Month	SLEDAI-2 K Score	Skin activity score
January	8 (4–13)	3 (1–7)
February	9 (5–11	2 (0–5)
March	8 (4–13)	2 (0–4)
April	6 (4–12)	4 (1–7)
May	9 (6–15)	5 (0–7)
June	13 (6–18)	8 (3–12)
July	12 (7–17)	7 (2–10)
August	12 (5–18)	8 (0–14)
September	10 (5–17)	3 (0–6)
October	8 (4–11)	2 (1–5)
November	7 (5–10)	2 (0–4)
December	7 (5–11)	2 (1–4)
*p* value	0.017*	0.0021**

^*^Using Bonferroni adjusted Mann Whitney U

^**^Using Bonferroni adjusted Mann Whitney U

## Discussion

In this study, we investigated the possibility of the influence of seasonal variation on the incidence of LN flare among Egyptian patients. The flares were assessed by month. Our data suggests clear statistically significant seasonal variation patterns of flares, with most occurring in the summer. The highest incidence of flares occurred in June (14%) and July (12.5%) (*p* = 0.003). The highest SLEDAI scores and skin activity scores were observed in summer months. In summertime, high temperature and ultraviolet rays increase which affect patients with season sensitive diseases. The recent climate changes in the form of greenhouse gas emission and global warming stand significant health hazards. Important international reports project that, by 2050, the climate changes will have health impacts; patients with climate sensitive diseases are most vulnerable.

Our study included 200 patients with LN flares maintaining regular follow-up at the Nephrology and Rheumatology clinics at Kasr Al-Ainy outpatient clinics. The diagnosis of lupus nephritis was confirmed by histopathological examination of kidney biopsy tissues. The median age for the patients is 33 years (IQR = 13). The median duration of lupus was 7 years. In our cohort, 92% of patients were females. The median serum creatinine was 1.4 mg/dl.

Multiple studies investigated lupus flare and its correlation with seasons. The seasonal pattern as demonstrated by our report agrees with a study by Chiche and colleagues [25], which included forty one SLE patients in southern France. They investigated the seasonal variation for the incidence of flare of cutaneous and non-cutaneous manifestations with visceral involvement of the disease and found a positive correlation between the occurrence of lupus flares and both temperature and the duration of sunshine increase. This pattern agrees with our data suggesting a possible link between warm climates and disease flares. Another study conducted on Chinese patients analyzed SLE flare patterns in 640 patients. Seasonal variations towards higher incidence in the winter were appreciated in that study [26]. Even though this pattern does not coincide with the summer flare patterns found in our study, several theories may explain it. Firstly, the study tested flare patterns of SLE and not specifically the activity of the LN. Secondly, they exclusively included patients of Chinese ethnicity, whereas our study cohort included an Egyptian population. A comparative study may be needed to determine whether ethnic backgrounds may play a role in disease activity and flares. Schlesingeran colleagues found an increased number of renal activities during spring and winter compared to summer and autumn [5].

Amit and colleagues [24] followed up 105 patients over 4 years and found no seasonal pattern of all SLE manifestations except photosensitivity, which increased in the summer. However, individual patients showed seasonal pattern of the disease activity [27]. For investigating the variations of flares, according to different seasons, they recorded the number of patients whose SLEDAI scores were higher than 12 and 18. This could have affected their results by missing patients with mild and moderate activity. Moreover, they only included renal patients of serum creatinine > 1.4, without assessment for proteinuria or active urine sediments. Their methodology was different from that used in our study.

The strength of our study includes maintained long-term regular follow-up, allowing early detection of LN flares by laboratory investigations. Moreover, we included a variety of LN stages, which mirror confirmed proportions of lupus nephritis incidence in SLE patients. Lastly, all patients were on antimalarial drugs and maintained on dual therapy for SLE.

The limitations of our study include its retrospective nature, as well as the difference in maintenance therapy, where 90% were receiving AZA and 10% were on MMF. We did not assess for patients’ education regarding the seasonal variations and the degree of weather protection they provided to themselves.

In conclusion, seasonal pattern of LN flare was observed in our study, with most flares observed during meteorological summertime. Larger studies are needed to confirm this seasonal pattern.
